# Care and Treatment of Epileptics

**Published:** 1901-03

**Authors:** 


**Care and Treatment of Epileptics.** By WILLIAM PRYOR LETCHWORTH, LL.D. Pp. xi., 246. New York: G. P. Putnam's Sons. 1900.

We recommend this book to those interested in the care and treatment of the sane and insane epileptic. Dr. Letchworth possesses a thorough knowledge of all that concerns this class of sufferers and that may minister to their comfort and improvement. Although the author has freely utilised a large collection of institution reports and statistics, he has by a happy facility of expression rendered his book agreeable enough reading. No little of the interest which the work excites is due to the evident, though unobtrusive, genuine philanthropic spirit in which it is written; it serves too as a grateful testimony of the benefit that has come from earnest endeavours to ameliorate the lot of an unfortunate class of humanity, and indirectly that of the community burdened by their existence if at large. The author reviews first the causes and principal features of epilepsy, and then the question of special provision and treatment. The greater portion of the book is occupied by well-selected accounts of the State and private institutions in North America and Europe, including England, for the care of the epileptic; details of construction and administration receive much attention. We do not learn anything definite as regards the medicinal and dietetic treatment of epilepsy; the majority of opinions, however, seems in favour of a very moderate meat diet and rare use of bromides. Greatest stress is laid upon open-air exercise and employment, healthy recreation and the fostering of altruistic tendencies in the patients. Dr. Letchworth has done well in placing this record of intelligent and kindly work before the public; there is much need for its general reading in this country, where a comparatively insignificant section of the sane epileptic class is treated in homes or colonies. A great number of weak-minded persons, epileptic or not, would be far better and more intelligently treated by being placed in properly adapted homes and colonies than by being drafted into asylums. We note progressive treatment in this direction at the Wakefield Asylum, and that Dr. Bevan Lewis is fully alive to the importance of the colony system. In course of time no doubt a general attempt will be made to separate the improvable from the irrecoverable portion of the insane class, as has been done at this institution. The book is well produced, and furnished with numerous illustrations.

